# Electrical and magnetic properties of antiferromagnetic semiconductor MnSi_2_N_4_ monolayer

**DOI:** 10.3389/fchem.2022.1103704

**Published:** 2022-12-08

**Authors:** Dongke Chen, Zhengyu Jiang, Ying Tang, Junlei Zhou, Yuzhou Gu, Jing-Jing He, Jiaren Yuan

**Affiliations:** ^1^ School of Physics and Materials Science Nanchang University, Nanchang, China; ^2^ School of Physics and Electronic Engineering, Jiangsu University, Zhenjiang, China; ^3^ College of Information Science and Technology, Nanjing Forestry University, Nanjing, China

**Keywords:** two-dimensional materials, antiferromagnetic semiconductor, half metals, electronic properties, biaxial strain

## Abstract

Two-dimensional antiferromagnetic semiconductors have triggered significant attention due to their unique physical properties and broad application. Based on first-principles calculations, a novel two-dimensional (2D) antiferromagnetic material MnSi_2_N_4_ monolayer is predicted. The calculation results show that the two-dimensional MnSi_2_N_4_ prefers an antiferromagnetic state with a small band gap of 0.26 eV. MnSi_2_N_4_ has strong antiferromagnetic coupling which can be effectively tuned under strain. Interestingly, the MnSi_2_N_4_ monolayer exhibits a half-metallic ferromagnetic properties under an external magnetic field, in which the spin-up electronic state displays a metallic property, while the spin-down electronic state exhibits a semiconducting characteristic. Therefore, 100% spin polarization can be achieved. Two-dimensional MnSi_2_N_4_ monolayer has potential application in the field of high-density information storage and spintronic devices.

## Introduction

In 2004, the successfully prepared graphene opened a new era of two-dimensional materials (Novoselov et al., 2004). Subsequently, numerous new systems have already been discovered, greatly promoting the development of the two-dimensional material family. Two-dimensional materials have a wide variety of electronic properties, including metallic, semi-metallic, semiconducting and insulating properties. For example, 1H-MoS_2_ is a semiconductor with a direct band gap, 1T phase MoS_2_ is a metal, while 1T’ phase MoS_2_ is semimetal (Hung et al., 2018). In addition, hexagonal boron nitride (h-BN) shows insulating properties (Liu et al., 2003), and graphene is semimetal (Sheng et al., 2019). However, many 2D materials lack intrinsic magnetism, such as graphene and MoS_2_, which motivates researchers to induce magnetism through defect engineering, adsorption or insertion of magnetic atoms. However, these schemes are difficult to construct stable long-range magnetic order. Therefore, two-dimensional intrinsic ferromagnetic materials have aroused tremendous attention.

According to Mermin-Wagner theory, the long-range magnetic order is predicted to be unstable in 2D material and can be easily destroyed by thermal fluctuations (Mermin and Wagner, 1966). Until 2017, the magnetism in the two-dimensional material CrI_3_ at the monolayer limit was observed experimentally (Gong et al., 2017; Huang et al., 2017). Hereafter, more 2D magnetic materials have been found, such as Fe_3_GeTe_2_ (Xian et al., 2022), FePS_3_ (Lee et al., 2016) and VSe_2_ (Bonilla et al., 2018). Two-dimensional magnetic materials possess a wide variety of excellent physical properties. For instance, monolayer magnetic metal materials have been widely used as electrodes in electronic devices, such as Fe_3_GeTe_2_ based van der Waals tunnel junctions (O’Hara et al., 2018). Furthermore, magnetic tunnel junction with antimagnetic semiconductor CrI_3_ tunnel barrier has been reported to possess a giant magnetoresistance effect due to the significant difference of energy band in the ferromagnetic and antiferromagnetic states (Song et al., 2018), which has achieved a huge breakthrough in spintronic devices. Hence antiferromagnetic semiconductor materials have become a hot research topic because of their novel band characteristics. However, such materials are very rare, the prediction of new antiferromagnetic semiconductor materials becomes the key to the development of spintronic devices.

In this paper, the electronic structure and magnetic properties of monolayer MnSi_2_N_4_ are explored based on first-principles calculations. The results demonstrate that 2D MnSi_2_N_4_ is a stable antiferromagnetic semiconductor in which the ground state is an antiferromagnetic state. The large magnetic exchange parameter indicates a strong antiferromagnetic coupling between the magnetic Mn atoms. When an external magnetic field is applied, the MnSi_2_N_4_ monolayer turns into a half-metal with a magnetic state transition from an antiferromagnetic state to a ferromagnetic state. In which the spin-up electronic state displays a metallic nature, while the spin-down electronic state exhibits a semiconducting feature. Therefore, the MnSi_2_N_4_ monolayer has great application prospects in spintronics and nanosensors.

## Computational details

All calculations were conducted using the Vienna *ab initio* simulation package (VASP) (Kresse and Furthmuller, 1996; Kresse and Joubert, 1999). The projection plane wave (PAW) method was adopted to describe the interaction between ions and electrons (Blöchl, 1994). The cutoff energy is set as 500 eV. The generalized gradient approximation (GGA) of the form Perdew–Burke–Ernzerhof (PBE) was employed to describe the exchange correlation (Perdew et al., 1996). The convergence criteria for electronic iteration and ionic relaxation were 10^–6^ eV and 0.001 eV/Å, respectively. An 18 Å vacuum layer was added in the out plane direction of the monolayer MnSi_2_N_4_ to eliminate interlayer interactions. The Brillouin zone was sampled with a 13 × 13 × 1 k-point mesh. Due to the strong correlation effect of Mn atoms, the DFT + U method proposed by Dudarev et al. (Dudarev et al., 1998) was adopted, and the effective parameter U_eff_ was set to 3.9 eV (Wang et al., 2006; Jain et al., 2011; Ling and Mizuno, 2012; Togo and Tanaka, 2015). The phonon spectrum of monolayer MnSi_2_N_4_ was calculated by the PHONONPY software (Togoet al., 2015) using a 5 × 5 supercell.

## Results and discussion

**FIGURE 1:**
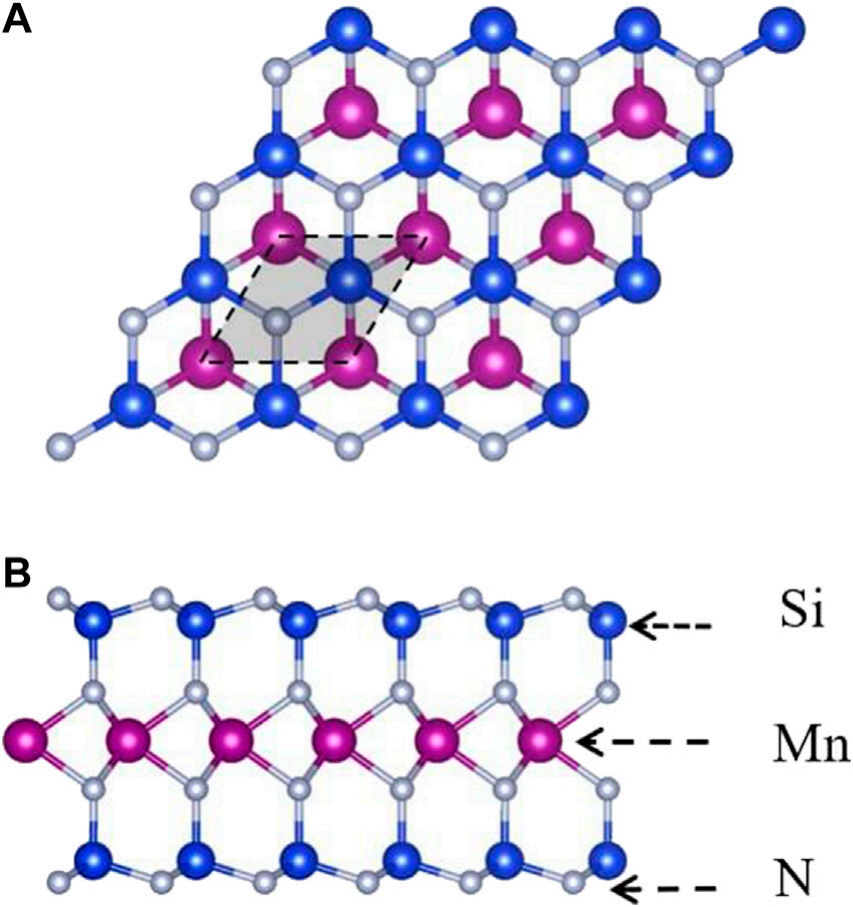
Top **(A)** and side **(B)** views of MnSi_2_N_4_. The balls are highlighted with blue (Si atom), purple (Mn atom) and silvery (N atom). The rhombic unit cells are marked with black dashed lines.

Similar to the two-dimensional MoSi_2_N_4_, the monolayer MnSi_2_N_4_ is a two-dimensional material with a hexagonal lattice structure and D_3h_ point group as shown in Figure 1. MnSi_2_N_4_ monolayer consists of seven atomic layers stacked with the order N-Si-N-Mn-N-Si-N, which can be regarded as a 1H-phase MnN_2_ triple-layer sandwiched between two buckled N-Si layers. The lattice constant of unit cell is 2.88Å, the bond length between Mn and N atoms is 2.02 Å and the bond length between Si and N atoms is 1.74 Å.

The cohesive energy of the monolayer MnSi_2_N_4_ was evaluated to confirm the stability of monolayer MnSi_2_N_4_ using the equation:

$$E_{coh}=\left(E_{Mn{Si}_{2}N_{4}}-E_{Mn}-2E_{Si}-4E_{N}\right)/7$$ (1)

**FIGURE 2:**
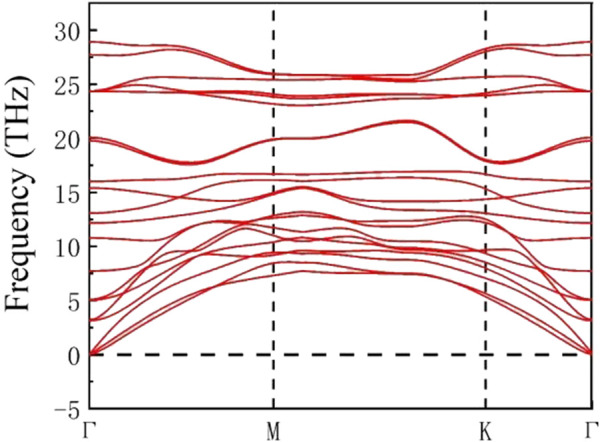
Phonon spectrum of monolayer MnSi_2_N_4._

Where $E_{Mn{Si}_{2}N_{4}}$ represents the energy of MnSi_2_N_4,_ $E_{Mn}$ , $E_{Si}$ and $E_{N}$ represent the energy of isolated single Mn, Si and N atoms, respectively. The calculated results show that the cohesive energy of MnSi_2_N_4_ is -5.03 eV/atom which is comparable to the value of MoS_2_ monolayer (−5.12 eV/atom) (Canton-Vitoria et al., 2020) and MoSi_2_N_4_ (−8.46 eV/atom) (Bafekry et al., 2021). We also calculated the phonon spectrum to check the stability, and there is no imaginary phonon frequency throughout the Brillouin zone, indicating that the structure is dynamically stable. Consequently, the MnSi_2_N_4_ monolayer has excellent stability and thus may be experimentally prepared in Figure 2.

**FIGURE 3:**
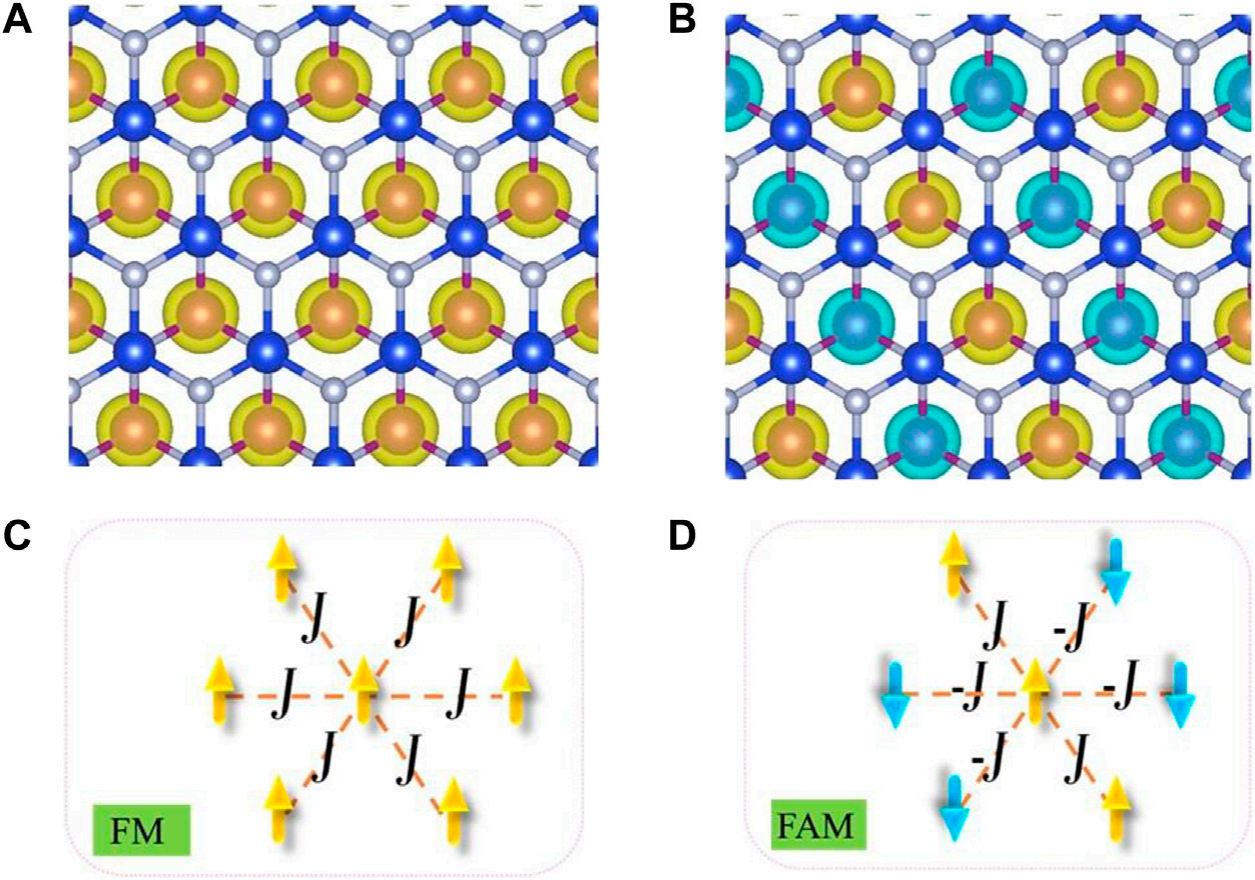
**(A,B)** spin-polarized charge densities of the FM and AFM case for MnSi_2_N_4_. The yellow and light blue isosurface with an isosurface value of 0.05 e/Bohr^3^ represent the spin-up and spin-down charge densities, respectively. **(C,D)** The scheme represents spin orientation of Mn atoms in FM and AFM case.

The magnetic properties of monolayer MnSi_2_N_4_ were investigated. We first determined the ground-state magnetic ordering with two possible magnetic order ferromagnetic (FM) and antiferromagnetic (AFM) states. The total energies of the AFM and FM phases of MnSi_2_N_4_ are -218.650eV and -217.658 eV, respectively. The energy of the AFM state is lower than that of the FM state, hence MnSi_2_N_4_ has an AFM ground state. The AFM order in monolayer MnSi_2_N_4_ sourced from the superexchange interactions between two magnetic atoms bridged by nonmetal atoms, following the Goodenough-Kanamori rules (Goodenough, 1955; Kanamori, 1959). In this case, the net magnetic moment is zero and the four Mn atoms in the supercell have an antiparallel magnetic state along with the same value of magnetic moments (3.05 μ_B_). The spin-polarized charge density and the schematic diagram for FM and AFM order are plotted in Figure 3. The spin-polarized charge density map shows that Mn atoms possess an high spin-polarized charge density, while spin-polariztion of N atoms is tiny with small magnetic moments (0.05 μ_B_).

**FIGURE 4:**
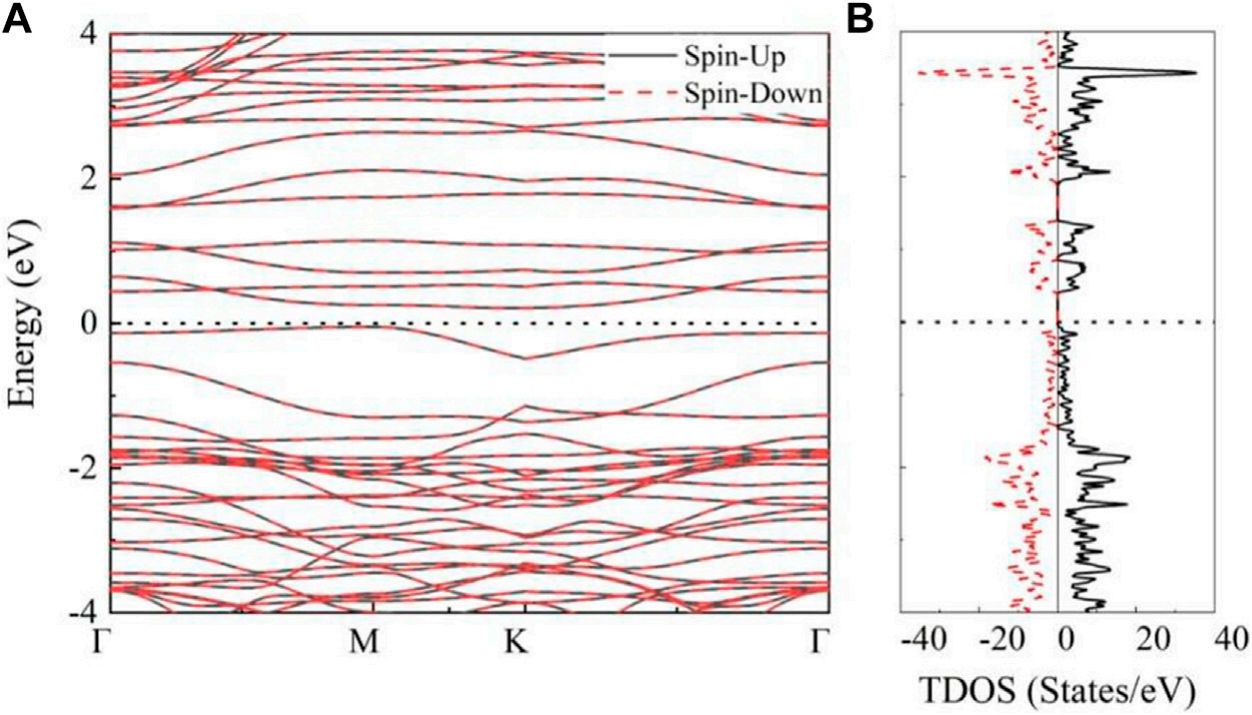
**(A)** The band structures and **(B)** total density of states (TDOS) of MnSi_2_N_4_ monolayer in the AFM states, the spin-up states and spin-down states are represented by the black lines and red dotted lines, respectively.

The electronic properties of AFM states are investigated to further explore potential applications of MnSi_2_N_4._ The electronic band structure and density of states (TDOS) are calculated as illustrated in Figure 4. It is clear that MnSi_2_N_4_ exhibits indirect semiconducting property without band cross Fermi level, which is different from nonmagnetic direct bandgap semiconductor MoSi_2_N_4_ monolayer (Yuan et al., 2022). The conduction band minimum (CBM) and the valence band maximum (VBM) are located at K point and M point, respectively. The band gap is small (0.26 eV). The bands are degenerate and the TDOS is symmetrical for spin-up and spin-down states. Furthermore, no states exist near the Fermi level along with a small energy gap.

**FIGURE 5:**
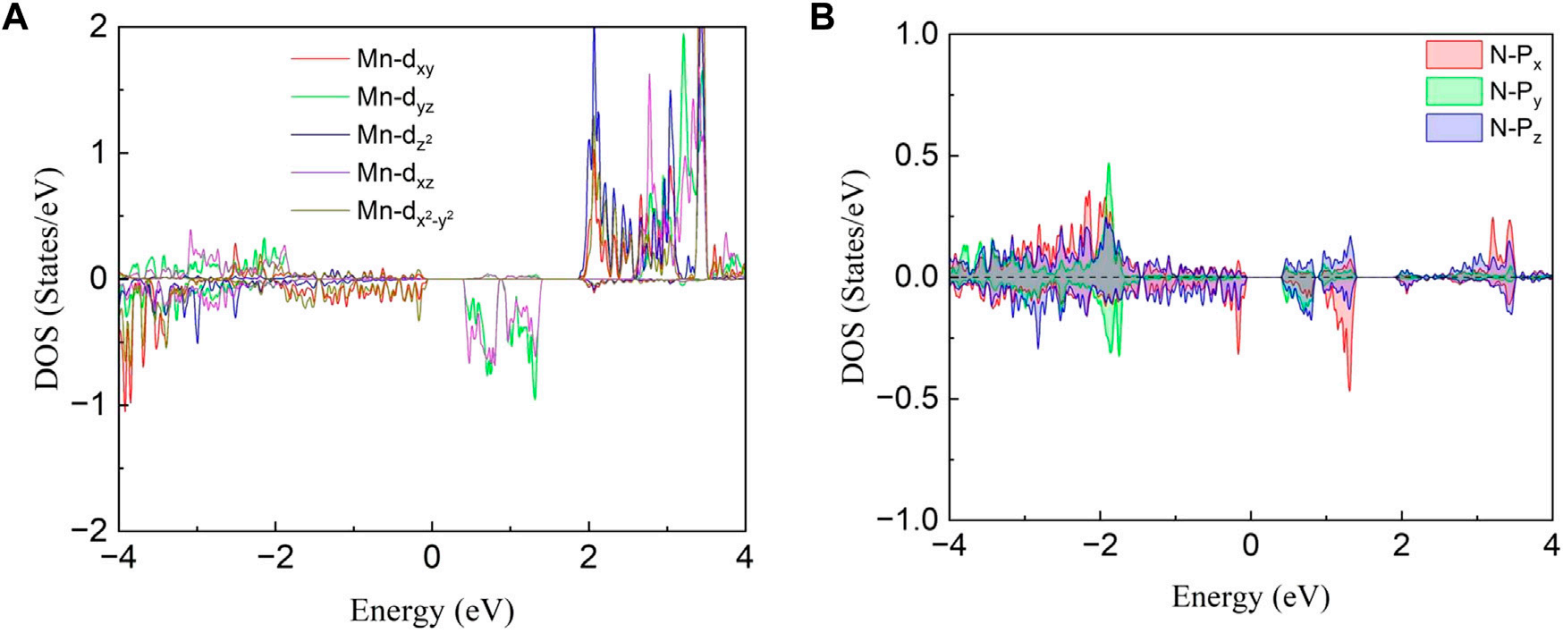
Projected density of states (PDOS) for **(A)** Mn-*d* orbital and **(B)** N-*p* orbital.

The projected density of states (PDOS) for Mn atom and the nearest neighbor N atom are depicted in Figure 5 to better analyze orbit contribution for electron structure and magnetic properties. One can notice that the density of states for the five 3*d* orbitals are all asymmetric as shown in Figure 5A, indicating the large spin splitting for an isolated Mn atom. The magnetic moment (3.04 μ_B_/Mn) is mainly dominated by the spin-up (majority-spin) states of *d* orbitals which is much more than the spin-down electron. For the N atom, the difference in PDOS between the spin-up and spin-down states is not obvious, resulting in a smaller magnetic moment. In addition, the DOS mainly comes from Mn-*d* and N-*p* in the energy range from 0.5 to 1.5 eV indicating that the hybridizations between the N-*p* and Mn-*d* orbitals are strong.

**FIGURE 6:**
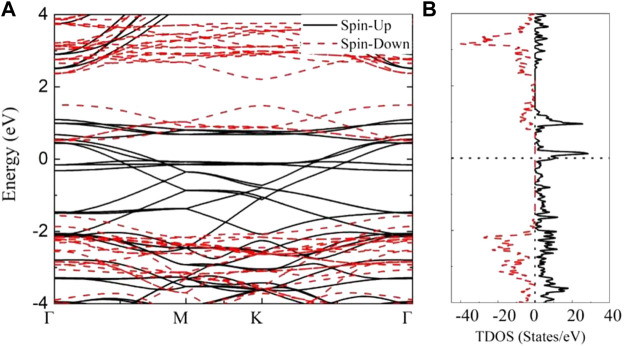
**(A)** The band structures and **(B)** total density of states (TDOS) of MnSi_2_N_4_ in the FM states, the spin-up states and spin-down states are represented by the black lines and red dotted lines, respectively.

Furthermore, the ground antiferromagnetic states will transition to ferromagnetic states under an external magnetic field. The band structure and density of states for FM state are depicted in Figure 6. It is clear that the spin-up and spin-down energy bands are not degenerate. The spin-polarized states can be noticed around the Fermi level. In the spin-up channel, several flat bands near the Fermi level existe and the others band display dispersion along Γ–M and K–Γ, which behave as a metal. But for the spin-down channel, a direct bandgap of 2.07 eV is observed with the VBM and CBM located at Γ point. Hence, MnSi_2_N_4_ monolayer behaves half-metallic properties in the FM case. The spin polarization is obvious with an asymmetric density of states distribution for spin-up and spin-down states as depicted in Figure 6B. One can find that a peak of spin-up states can be seen near the Fermi level and a large bandgap exists in the spin-down states, which further confirms the metallic behavior for the spin-up states and semiconducting property for spin-down states, respectively. Therefore, the 2D MnSi_2_N_4_ in FM state is a half-metal with 100% spin polarization.

**FIGURE 7:**
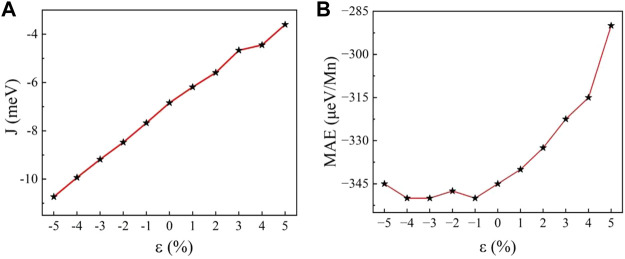
**(A)** Magnetic exchange parameter J and **(B)** magnetocrystalline anisotropy energy *E*_MAE_ as a function of strain.

Strain is an effective means of manipulating electronic structure and magnetic properties which is widely utilized to modulate the electronic structure and magnetic properties of monolayer system. In this paper, the strain is defined as *ε* = (a − a_0_)/a_0,_ where a_0_ is the relaxed lattice constant in the equilibrium state. The magnetic moment of the Mn atom remains about 3 μ_B_ per unit cell under strain. The effective spin Hamiltonian based on the Heisenberg model can be expressed as

$$H=-\underset{<i,j>}{\sum }J_{ij}\mu_{i}\mu_{j}$$ (2)

where $J_{ij}$ is the magnetic exchange parameter, and $\mu_{i}/\mu_{j}$ is the magnetic moment at nearest neighbor sites i and j, respectively (Kan et al., 2013). The magnetic exchange parameter *J* is a significant parameter, which can be evaluated by calculating the total energy of the system in different magnetic states. For the FM case, the total energy can be written as *E*_FM_ = *E*_0_ − 3 *J* | μ |^2^, where *E*_0_ represents the total energy without spin polarization. For the AFM case, the total energy can be expressed as *E*_AFM_ = *E*_0_ + J | μ |^2^. Thus, the exchange parameter can be extracted by *J* = (*E*_AFM_ − *E*_FM_)/4 | μ |^2^. According to Figure 7A, although the value of *J* increases nearly linearly with biaxial strain, the energy difference between AFM case and FM case remains positive over the range of applied biaxial strain, indicating that MnSi_2_N_4_ behaves as AFM phase and no transition from AFM to FM phase is observed. Furthermore, the magnetic exchange parameter J increases with tensile strain and decreases with compressive strain. According to this trend, an extremely large tensile strain may be needed to turn the AFM to the FM ordering.

To identify the easy axis of MnSi_2_N_4_, we computed the magnetic anisotropy energy (MAE). The MAE of the magnetic crystal is defined as *E*_MAE_ = *E*_in_-*E*_out_ (Webster and Yan, 2018), that is, the energy difference between the in-plane (*E*_in_) and out-of-plane (*E*_out_) of MnSi_2_N_4_. For the strain-free monolayer MnSi_2_N_4_, the MAE is −345 μeV/Mn atom, indicating that the easy axis of MnSi_2_N_4_ prefers in-plane and the spin of the Mn atoms is arranged parallel to the basal plane. The MAE of monolayer MnSi_2_N_4_ is mainly derived from Mn atoms since Mn atoms have relatively stronger spin-orbit coupling than other atoms. The MAE is depicted as a function of strain in Figure 7B. When the structure is compressed, this value fluctuates around −350 μeV/Mn, hence the effect of compressive strain on MAE is not obvious. While MAE increases significantly with increasing tensile strain. MAE changes from -345 μeV/Mn to −290 μeV/Mn under the 5% tensile strain.

## Conclusion

The electronic and magnetic properties of monolayer MnSi_2_N_4_ are explored based on first-principles calculations. Monolayer MnSi_2_N_4_ is an intrinsic antiferromagnetic semiconductor with a small indirect band gap (0.26 eV). The MnSi_2_N_4_ has strong antiferromagnetic coupling along with strong in-plane magnetocrystalline anisotropy energy (−345 μeV/Mn). Furthermore, the MnSi_2_N_4_ monolayer exhibits half-metallic properties with a metallic spin-up state and a semiconducting spin-down state. The effect of biaxial strain on magnetism is also investigated. The magnetic exchange parameter J and MAE increase with biaxial tensile strain. The tunable magnetic properties may enrich the 2D antiferromagnets community and stimulate potential applications in spintronic devices.

## Data Availability

The original contributions presented in the study are included in the article/Supplementary Material, further inquiries can be directed to the corresponding author.
